# Covid-19: A new global threat for future generations

**DOI:** 10.18502/ijrm.v19i2.8478

**Published:** 2021-02-21

##  Dear Editor,

SARS-CoV-2 is a single-stranded RNA virus that acts through angiotensin-converting enzyme 2 (ACE2) as its main receptor. ACE2 expression has been revealed to be distributed in many organs of the human testis such as Leydig and Sertoli cells. Also, this virus can induce oxidative stress and increase free radicals in these cells. This event facilitates sperm DNA fragmentation that leads to reducing sperm motility and viability. As has been demonstrated, Leydig and Sertoli cells are responsible for influencing testosterone production and spermatogenesis. Angiotensin 1-7 is produced by ACE2 in the testicular tissues that activate through the Mas receptor and increase sperm motility via Phosphatidylinositol 3-kinase (PI3K)/protein kinase B (Akt) signaling pathway. In COVID-19, the expression of ACE2 in human cells is reduced. So, the Ang (1-7)/Mas/PI3K/AKT3 pathway is destroyed in the testis. This alteration in turn may decrease spermatogenesis, sperm motility, and male fertility. Moreover, this virus has been detected in the patients' semen 36 days after recovery. SARS-CoV-2 induce hyper-inflammatory syndrome by increasing inflammatory cytokines' actions through the ACE2 receptor. This event can produce pulmonary, distal alveoli, pulp, and irreversible damages in other organs that can further cause the cell apoptosis and multi-organ system failure (1-8). Therefore, due to the irreversible damage of SARS-CoV-2 to other organs such as the lungs through a mechanism similar to the mechanism of action in the testis and the long-term presence of that in the semen or male reproductive system, it can be suggested that this virus can induce male reproductive disorders in patients with COVID-19 in future. Hence, male infertility in patients with COVID-19 should be considered as a serious problem in the future generation that will require extensive clinical research to clarify its related problems and ways to treat them.

##  Conflict of Interest 

None declared.

**Ali Akbar Oroojan Ph.D.**

Department of Physiology, Faculty of Medicine, Dezful University of Medical Sciences, Dezful, Iran.
